# Anesthetic Approaches and Their Impact on Cancer Recurrence and Metastasis: A Comprehensive Review

**DOI:** 10.3390/cancers16244269

**Published:** 2024-12-22

**Authors:** Hoon Choi, Wonjung Hwang

**Affiliations:** Department of Anesthesiology and Pain Medicine, Seoul St. Mary’s Hospital, College of Medicine, The Catholic University of Korea, Seoul 06591, Republic of Korea; hoonie83@catholic.ac.kr

**Keywords:** anesthesia, cancer recurrence, metastasis, perioperative care

## Abstract

Cancer recurrence and metastasis remain critical challenges following surgical resection, influenced by complex perioperative mechanisms. This review explores how surgical stress triggers systemic changes, such as neuroendocrine responses, immune suppression, and inflammation, which promote the dissemination of residual cancer cells and circulating tumor cells. Key mechanisms, such as epithelial–mesenchymal transition and angiogenesis, further enhance metastasis, while hypoxia-inducible factors and inflammatory responses create a microenvironment conducive to tumor progression. Anesthetic agents and techniques modulate these mechanisms in distinct ways. Inhaled anesthetics, such as sevoflurane, may suppress immune function by increasing catecholamines and cytokines, thereby promoting cancer progression. In contrast, propofol-based total intravenous anesthesia mitigates stress responses and preserves natural killer cell activity, supporting immune function. Opioids suppress immune surveillance and promote angiogenesis through the activation of the mu-opioid receptor. Opioid-sparing strategies using NSAIDs show potential in preserving immune function and reducing recurrence risk. Regional anesthesia offers benefits by reducing systemic stress and immune suppression, though the clinical outcomes remain inconsistent. Additionally, dexmedetomidine and ketamine exhibit dual effects, both enhancing and inhibiting tumor progression depending on the dosage and context. This review emphasizes the importance of individualized anesthetic strategies to optimize long-term cancer outcomes. While retrospective studies suggest potential benefits of propofol-based total intravenous anesthesia and regional anesthesia, further large-scale trials are essential to establish the definitive role of anesthetic management in cancer recurrence and survival.

## Simple Summary

Cancer recurrence and metastasis are significant challenges following surgery. Recent research suggests that anesthetic techniques used during cancer surgery may influence oncologic outcomes by affecting the body’s immune response, stress levels, and inflammation. This review explores how different anesthetic methods may modulate perioperative pathophysiologic changes. Although the early findings are promising, the impact of anesthesia on long-term cancer outcomes remains unclear. Ongoing clinical trials aim to provide clearer insights into the impact of anesthesia, particularly in lung, breast, and colorectal cancer surgeries. These efforts highlight the importance of tailoring anesthetic care to meet the specific needs of individual patients. As more evidence emerges, it could guide the development of improved anesthetic strategies that not only enhance recovery but also reduce the risk of cancer recurrence.

## 1. Introduction

Cancer recurrence and metastasis remain critical challenges in the management of cancer patients, often resulting in poor long-term outcomes despite advancements in surgical and systemic therapies. The perioperative period represents a crucial window during which various physiological and molecular changes can influence cancer progression [1,2]. Factors such as neuroendocrine stress responses, immune suppression, and systemic inflammation during this time have been implicated in promoting the survival and dissemination of residual cancer cells [1,3].

Anesthetic management, traditionally centered on maintaining intraoperative stability and safety, has increasingly been recognized for its potential impact on oncologic outcomes [4,5,6]. Different anesthetic agents and techniques exert varying effects on surgical stress, immune function, and inflammatory pathways [7,8]. Recent studies have also identified key mechanisms, such as angiogenesis, epithelial–mesenchymal transition (EMT), and immune evasion, which are influenced by anesthetic management [9,10]. A deeper understanding of these interactions could guide the development of tailored perioperative strategies to optimize cancer care.

Although numerous studies have explored the relationship between anesthetic management and cancer outcomes, the findings remain fragmented and inconsistent due to heterogeneity in study design, cancer types, and evaluation markers. This review aims to synthesize the existing evidence, offering a comprehensive overview of how anesthetic agents and techniques may influence cancer recurrence and metastasis. By integrating insights from clinical trials and mechanistic studies, it proposes to offer a new perspective on perioperative management in cancer patients, highlighting potential strategies to improve both immediate surgical outcomes and long-term survival.

## 2. Materials and Methods

A comprehensive literature search was conducted using PubMed, MEDLINE, Google Scholar, and the Cochrane Library to identify relevant studies published up to December 2023 on the relationship between anesthetic management and cancer recurrence. The search terms included combinations of the following keywords: “anesthesia”, “cancer recurrence”, “metastasis”, “general anesthesia”, “regional anesthesia”, “volatile anesthetics”, “intravenous anesthetics”, “opioid agents”, “non-opioid agents”, and “local anesthetics”.

**Figure 1:**
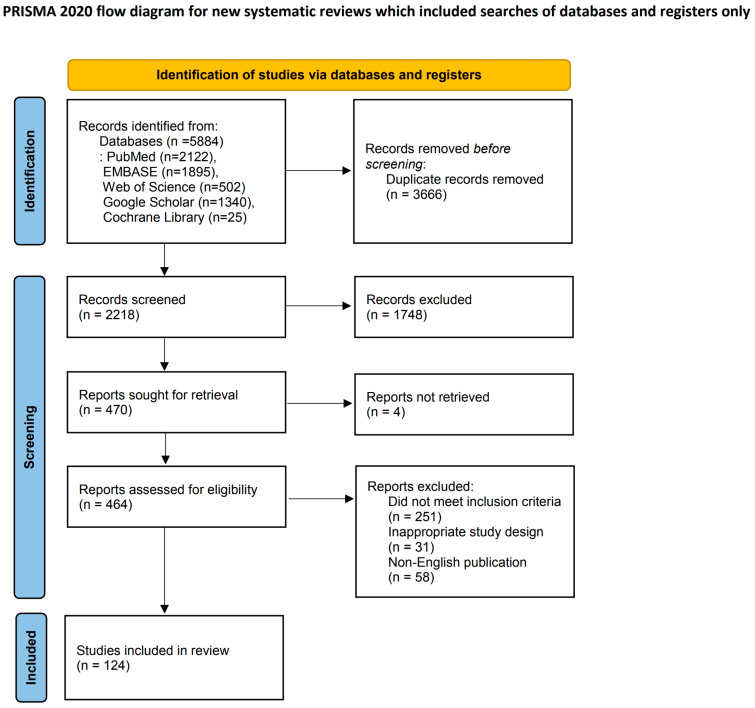
PRISMA flow diagram.

We adhered to the PRISMA (Preferred Reporting Items for Systematic Reviews and Meta-Analyses) guidelines in identifying, screening, and selecting studies (Figure 1). All the retrieved articles were manually examined, and additional studies were identified by screening the reference lists of relevant reviews and articles. Articles were selected based on their relevance to the research focus, specifically examining the impact of various anesthetic agents and techniques on cancer recurrence and metastasis across different cancer types.

Eligible studies included original research articles, meta-analyses, systematic reviews, and clinical trials published in peer-reviewed journals. Preclinical studies were included only if they provided substantial insights into mechanisms relevant to clinical outcomes. Studies were excluded if they were non-English publications, case reports, or focused on topics unrelated to anesthesia or cancer recurrence and metastasis. Articles that solely addressed other treatment modalities, such as chemotherapy or radiotherapy, without specific reference to anesthetic management were also excluded.

## 3. Surgical Contributions to Cancer Recurrence and Metastasis: Cellular, Microenvironmental, and Systemic Changes

**Figure 2:**
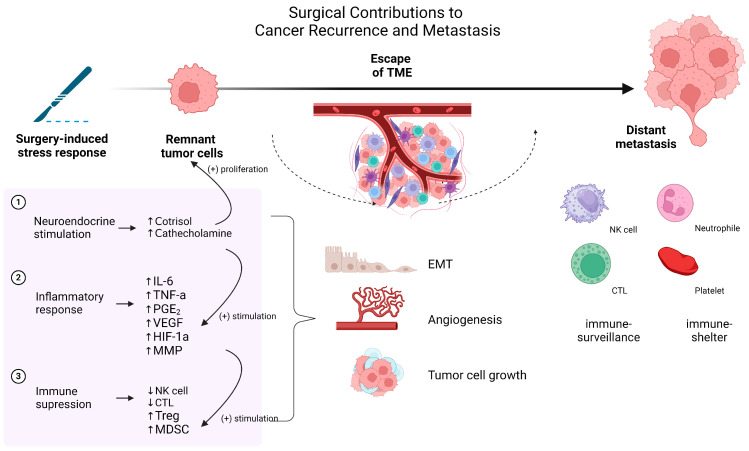
Surgical contributions to cancer recurrence and metastasis: interaction between tumor microenvironment and systemic responses. IL-6: interleukin-6, TNF-α: tumor necrosis factor-alpha, PGE_2_: prostaglandin E_2_, VEGF: vascular endothelial growth factor, HIF-1α: hypoxia-inducible factor-1 alpha, MMP: matrix metalloproteinase, NK cell: natural killer cell, CTL: cytotoxic T lymphocyte, Treg: regulatory T cell, MDSC: myeloid-derived suppressor cell, TME: tumor microenvironment, EMT: epithelial–mesenchymal transition.

Surgical intervention, although a critical component of cancer treatment, can paradoxically contribute to tumor recurrence and metastasis through several biological mechanisms [1,2]. Removal of the primary tumor often leaves behind residual cancer cells, which interact with the tumor microenvironment (TME) to promote further malignancy. Moreover, surgery can facilitate the release of circulating tumor cells (CTCs) into the bloodstream, increasing the risk of distant metastasis. Systemic responses to surgical stress, including inflammation, immunosuppression, and hormonal fluctuations, can compromise the body’s defense systems, allowing residual cancer cells to survive, proliferate, and spread [4,11]. Figure 2 provides a comprehensive schematic representation of these processes, highlighting the key mechanisms through which surgical stress contributes to cancer recurrence and metastasis.

### 3.1. Residual Cancer Cells and Their Microenvironmental Interactions

Despite curative intent, microscopic clusters of cancer cells frequently remain at the surgical margins or within the lymphovascular system following resection [11]. These residual cancer cells can invade surrounding tissues, spread through lymphatic or vascular channels, or disseminate into body cavities, such as the peritoneum or pleura. Surgical manipulation can exacerbate these processes by disrupting tissue integrity and facilitating the release of cancer cells into circulation.

CTCs are a key driver of distant metastasis and are commonly detected in patients with solid tumors [12]. Their numbers often increase following surgery, correlating with poorer prognoses [13,14,15]. However, not all CTCs successfully establish metastatic sites; only those capable of evading immune surveillance, particularly during the postoperative period of heightened immune suppression and inflammation, have the potential to form metastatic colonies [12].

Residual cancer cells reside within a complex TME consisting of non-malignant cells, extracellular matrix components, and various signaling molecules [16,17]. The TME plays a crucial role in both local recurrence and distant metastasis. Surgical stress can significantly alter the TME, creating conditions that favor cancer cell survival, invasion, and dissemination.

EMT is a critical mechanism enabling cancer cell dissemination [16,18]. During EMT, cancer cells lose their epithelial characteristics and acquire mesenchymal properties, facilitating tissue invasion and entry into the bloodstream or lymphatics. Once in circulation, CTCs encounter immune defenses, particularly attacks from natural killer (NK) cells and cytotoxic T lymphocytes (CTLs).

Postoperative immunosuppression and systemic inflammatory responses further impair immune function, facilitating CTC survival and metastasis [13,14]. CTCs that evade immune surveillance may lodge in microvascular beds of distant organs, where they initiate metastatic colonies.

### 3.2. Systemic Responses After Surgery and Cancer Recurrence and Metastasis

Surgical interventions trigger a cascade of systemic changes, influencing cancer recurrence by disrupting neuroendocrine function, immune responses, and inflammatory pathways [1,2]. These alterations foster an environment where residual cancer cells can evade immune surveillance and establish metastatic foci.

#### 3.2.1. Neuroendocrine and Inflammatory Modulation

Surgical trauma induces a significant neuroendocrine response by releasing catecholamines and glucocorticoids [11]. This surge in stress hormones affects metabolic processes and has profound effects on immune and inflammatory pathways. Catecholamines bind to β-receptors on tumor cells, promoting EMT, a critical process in cancer cell invasion and metastasis [19]. Additionally, catecholamines stimulate the release of pro-inflammatory cytokines, such as interleukin (IL)-6 and IL-8, as well as vascular endothelial growth factor (VEGF), which can enhance angiogenesis and tumor cell dissemination [20,21].

The inflammatory response, while essential for tissue repair, can become pathological in the context of cancer. Neutrophils and macrophages initiate the inflammatory response by releasing pro-inflammatory cytokines, like IL-1, IL-6, and tumor necrosis factor (TNF)-α. These immune cells also secrete proteases and matrix metalloproteinases (MMPs) that degrade the extracellular matrix, facilitating cancer cell invasion [22,23]. Neutrophil extracellular traps (NETs), produced by activated neutrophils, contribute to cancer progression by capturing CTCs and promoting their adhesion to endothelial cells [24,25]. IL-6 triggers macrophages to release prostaglandin E_2_ (PGE_2_), which creates a cytokine imbalance and suppresses cell-mediated immunity [26]. PGE_2_ also enhances tumor cell migration and angiogenesis, supporting metastasis.

Surgical stress exacerbates cytokine imbalances, increasing immunosuppression [1,27]. Pro-inflammatory cytokines decrease, while anti-inflammatory cytokines increase, promoting the expansion of immunosuppressive regulatory T (Treg) cells and helper T (Th)2 cells and reducing CTLs and Th1 cells. Additionally, fibroblasts and mesenchymal cells promote cancer progression by secreting growth factors (VEGF, epidermal growth factor (EGF)), enzymes (MMPs, cyclooxygenase [COX]-2), and signaling molecules that further enhance tumor growth and metastasis [3,23].

#### 3.2.2. Immunosuppression and Tumor Escape Mechanisms

Surgery-induced immunosuppression allows residual cancer cells to evade immune detection and proliferate [1,28]. Immediately after surgery, the function of NK cells and CTLs, critical for identifying and eliminating tumor cells, is significantly diminished. This reduction in cytotoxic activity enables CTCs and residual tumor cells to escape immunosurveillance, increasing the likelihood of metastasis to distant sites.

Tregs and myeloid-derived suppressor cells (MDSCs) further suppress anti-tumor immunity. Tregs inhibit NK cells and CTLs’ function, creating an environment that favors tumor progression [1,12]. MDSCs enhance tumor growth by promoting angiogenesis and establishing premetastatic niches, which provide a supportive environment for tumor cell survival [29]. Elevated levels of Tregs and MDSCs are associated with worse outcomes and higher recurrence rates in several cancers [30,31,32].

Hypoxic conditions resulting from surgical trauma further exacerbate these effects by stabilizing hypoxia-inducible factor (HIF)-1α [33,34]. HIF-1α stimulates the expression of VEGF and other pro-angiogenic factors, promoting tumor growth and metastasis. The combination of immune suppression, inflammatory dysregulation, and hypoxia creates a postoperative microenvironment that favors cancer recurrence. Overexpression of HIF-1α and VEGF has been linked to poor prognosis in several cancers [35,36].

#### 3.2.3. Interactions Between Cancer Cells and Systemic Factors

Once CTCs detach from the primary tumor and enter the bloodstream, they encounter systemic factors that can either enhance or hinder their survival and dissemination. Inflammatory mediators, including cytokines and chemokines, significantly influence the metastatic potential of CTC metastasis [22,23,24]. For example, elevated levels of IL-6 after surgery promote EMT, enhancing the invasiveness of CTCs. NETs, formed in response to systemic inflammation, capture CTCs, aiding their adhesion and migration to distant organs. These processes underscore the complex interplay between cancer cells and the systemic changes induced by surgery.

Platelets play a pivotal role in aiding CTCs. By forming aggregates with CTCs, platelets shield them from immune detection and protect them from the physical forces in circulation [37]. This interaction allows CTCs to survive in the bloodstream and adhere to endothelial cells at distant sites, promoting metastasis. Moreover, activated platelets secrete growth factors, such as platelet-derived growth factor and transforming growth factor (TGF)-β, which remodel the TME and promote cancer cell proliferation and invasion [38]. Perioperative increases in platelet levels have been associated with a poor cancer prognosis [39].

Fibrinogen and complement activation also contribute to CTC survival. Fibrinogen forms protective complexes around CTCs, helping them evade immune detection [40]. Complement activation stimulates pro-angiogenic and immunosuppressive pathways, further promoting metastasis [41,42,43]. These systemic interactions between CTCs, platelets, and inflammatory factors are essential in determining whether residual tumor cells successfully colonize distant organs, leading to cancer recurrence.

## 4. Impact of Anesthetic Management on Cancer Recurrence

Given the significant influence of perioperative factors on tumor progression, optimizing anesthetic strategies is essential for improving patient outcomes. This section reviews commonly used anesthetic agents and techniques in cancer surgeries, focusing on their effects on stress responses, inflammatory pathways, and immune modulation, as well as their potential role in cancer recurrence and metastasis (Table 1). Relevant clinical studies are also summarized to understand the complex interactions between anesthesia and oncologic outcomes comprehensively (Table 2).

### 4.1. Inhaled Anesthetics vs. Propofol-Based Total Intravenous Anesthesia (TIVA)

#### 4.1.1. Mechanistic and Preclinical Insights

Inhaled anesthetics, such as isoflurane and sevoflurane, stimulate the hypothalamic–pituitary–adrenal axis, leading to increased release of catecholamines and cortisol, which suppress immune responses by reducing NK cell and CTL activity [133,134]. Elevated stress hormones further promote the release of pro-inflammatory cytokines, such as IL-6 and TNF-α, which enhance tumor proliferation and migration. In preclinical models, inhaled anesthetics have been shown to upregulate HIF-1α and VEGF, promoting angiogenesis and cancer cell survival [7,44,45,46]. In breast, lung, and ovarian cancer models, inhaled anesthetics enhance CTC survival and tumor invasiveness [47,48,49,50,51,52]. However, some studies suggest that high concentrations of sevoflurane may inhibit tumor invasion by downregulating the *p*-38 MAPK pathway, reducing HIF-1α activation and suppressing MMP-2/-9 activity [48,135,136,137]. These conflicting results may arise from variations in experimental conditions, including differences in cell lines, anesthetic exposure duration, and concentration levels, some of which exceed clinical relevance.

In contrast, propofol demonstrates antitumor effects through its ability to reduce stress hormones and pro-inflammatory cytokines, including IL-6 and PGE_2_, while enhancing NK cell cytotoxicity and maintaining a favorable Th1/Th2 balance [10,138,139,140,141]. Propofol also inhibits tumor angiogenesis by downregulating HIF-1α and VEGF [45,53,54] and suppresses tumor migration and invasion through the ERK 1/2 pathway and MMPs [55,56,57]. Preclinical studies consistently show that propofol induces cancer cell apoptosis via the p53 pathway and reduces metastatic potential across various cancer models [45,53,54].

#### 4.1.2. Clinical Findings and Outcomes

Retrospective studies generally favor propofol-based anesthesia over inhaled anesthetics for oncologic outcomes. A large study by Wigmore et al., involving over 7000 patients, demonstrated a significant survival benefit with propofol-based TIVA compared to inhaled anesthetics [129]. In a propensity-matched analysis, the 5-year mortality rate was 15.6% in the TIVA group versus 22.8% in the inhaled anesthetics group (hazard ratio (HR) 1.46, 95% confidence interval (CI) 1.29–1.66, *p* < 0.001), with the greatest benefit observed in gastrointestinal malignancies. Similarly, a retrospective analysis of 2856 gastrointestinal cancer patients reported improved overall survival (OS) with propofol (HR 0.65, 95% CI 0.56–0.75, *p* < 0.001) [115]. Another study on hepatic cancer showed better survival outcomes with propofol, regardless of tumor stage or metastasis status [116]. Other studies also demonstrated improved OS and recurrence-free survival (RFS) in gastrointestinal and hepatic cancers [117,118].

However, the benefits of propofol are less consistent in other cancer types. Studies on lung and brain cancers have not shown clear survival advantages [108,127,128]. In breast cancer, the retrospective findings are mixed. A study of 6305 breast cancer patients showed a survival advantage with propofol over sevoflurane, with a maximum 5-year OS difference of 9.2% depending on the statistical adjustment method (HR 1.46, 95% CI 1.10–1.95) [99]. Another analysis of 325 breast cancer patients undergoing modified radical mastectomy found that propofol significantly reduced the recurrence rates compared to sevoflurane (HR 0.55, 95% CI 0.311–0.973, *p* = 0.037), although the OS did not differ between the two groups [100]. Several other retrospective studies found no significant differences in recurrence or survival between the two anesthetic approaches [101,102,103].

Randomized controlled trials (RCTs) have yielded similarly mixed results. The Cancer and Anesthesia Study also found no significant difference in 1-year survival between propofol and volatile anesthetics in breast cancer [142]. Another RCT showed that propofol reduced local recurrence in ductal carcinoma but had no impact on metastatic progression [104].

Meta-analyses have added complexity to the debate. A review by Chang et al. demonstrated that propofol-based TIVA improved OS across various cancers (HR 0.79, 95% CI 0.66–0.94, *p* = 0.008), with the most significant benefit observed in gastrointestinal cancers [130]. However, no significant difference in RFS was reported (HR 0.81, 95% CI 0.61–1.07, *p* = 0.137). A subgroup analysis further revealed greater OS benefits for propofol compared to desflurane (HR 0.54, 95% CI 0.36–0.80, *p* = 0.003) but no significant difference compared to sevoflurane (HR 0.92, 95% CI 0.74–1.14, *p* = 0.439). Yap et al. similarly reported improved OS and RFS with TIVA in breast, esophageal, and non-small-cell lung cancers (NSCLC) (pooled HR 0.78, 95% CI 0.65–0.94, *p* < 0.01) [131]. However, a large retrospective study of over 196,000 gastrointestinal cancer patients found no significant differences in either OS or RFS between TIVA and inhaled anesthetics (HR for OS, 1.02; 95% CI, 0.98–1.07; *p* = 0.28; HR for RFS, 0.99; 95% CI, 0.96–1.03; *p* = 0.59) [114].

In conclusion, while retrospective data often support the oncologic benefits of propofol, the results from RCTs and meta-analyses remain inconsistent. The discrepancies in these findings may be attributed to variations in study design, cancer types, patient populations, and statistical methods. Propofol shows more consistent benefits in gastrointestinal and hepatic cancers, while results in lung, brain, and breast cancers are mixed. Further large-scale trials are needed to clarify the definitive impact of anesthetic choice on cancer outcomes.

### 4.2. Opioids

#### 4.2.1. Mechanistic and Preclinical Insights

Opioids, such as morphine and fentanyl, are widely used for pain management in cancer surgeries but may have unintended oncologic effects. They suppress innate immunity indirectly by activating the sympathetic nervous system, leading to increased catecholamine and cortisol levels, which in turn inhibit NK cell and CTL activity [143,144]. Additionally, opioids directly modulate immune responses through their interaction with μ-opioid receptors (MOR) on immune cells, impairing NK cell function and promoting angiogenesis [145,146,147]. MOR activation facilitates cancer cell migration, invasion, and angiogenesis by activating pathways such as ERK and PI3K [144,148,149].

Preclinical studies consistently demonstrate these effects. In vitro studies show that morphine reduces NK cell activity, impairing immune surveillance and facilitating metastasis [143,144]. Opioids also promote angiogenesis by upregulating VEGF, enhancing tumor growth [9]. In animal models of lung and breast cancer, morphine has been shown to impair NK cell function and increase metastasis via VEGF-driven angiogenesis [58,59,61,62]. Furthermore, MOR overexpression on cancer cells amplifies these tumor-promoting effects [58,60,61,148,149].

Conversely, opioid antagonists, like methylnaltrexone and naloxone, have shown anti-tumor effects in preclinical models. In NSCLC and breast cancer studies, these antagonists reduced cancer cell proliferation and metastasis, suggesting that blocking opioid receptors may inhibit tumor progression [150,151]. Interestingly, tramadol, a unique opioid, has exhibited immune-stimulating effects by enhancing NK cell cytotoxicity in preclinical studies, indicating a potential protective role under specific conditions [152].

#### 4.2.2. Clinical Findings and Outcomes

Retrospective studies have investigated the relationship between perioperative opioid use and cancer outcomes, but the results remain inconsistent. In NSCLC, a retrospective study found that high intraoperative doses of fentanyl were associated with decreased OS in Stage I patients (*p* = 0.036), with no significant impact observed in Stage II or III patients [109]. Similarly, no effect on RFS was found across any stage, although the Stage I patients showed a trend toward significance for opioid use as a risk factor (*p* = 0.053). Another small retrospective study revealed that higher postoperative opioid consumption was linked to increased recurrence rates in NSCLC, with the recurrence group receiving significantly higher total opioid doses over 96 h postoperatively (232 mg vs. 124 mg morphine equivalents, *p* = 0.02) [110]. However, other studies have failed to show a significant association between perioperative opioid dose and cancer recurrence or survival in lung cancer patients [111].

In colorectal cancer, a retrospective study reported no correlation between intraoperative fentanyl dose and either RFS or OS [153]. Similarly, studies involving esophageal cancer patients presented conflicting results on the impact of perioperative opioid use on oncologic outcomes [119,120,121]. These discrepancies suggest that the oncologic effects of opioids may depend on factors such as cancer type, opioid dose, and individual patient characteristics.

Meta-analyses also provide mixed findings. While they generally found no consistent evidence that opioids significantly affect cancer recurrence in animal or human studies, the data were limited and heterogeneous, making definitive conclusions challenging [154,155]. These analyses were based on limited and heterogeneous data, making it difficult to draw definitive conclusions.

In summary, opioids remain essential for managing perioperative pain in cancer patients but concerns about their potential immunosuppressive and tumor-promoting effects persist. While clinical evidence suggests that the type and dosage of opioids may influence cancer recurrence and progression, the findings remain inconclusive. Large-scale RCTs are needed to better understand opioids’ role in cancer surgery and to identify optimal analgesic strategies that minimize recurrence while ensuring effective pain control.

### 4.3. Non-Opioid Adjuvants

#### 4.3.1. NSAIDS

(1) Mechanistic and Preclinical Insights

NSAIDs are of interest in cancer surgery for their anti-inflammatory properties, primarily mediated through COX inhibition, particularly COX-2, which reduces PGE2 production [63,64]. Elevated PGE2 levels contribute to cancer progression by stimulating angiogenesis, promoting tumor cell proliferation, and inhibiting apoptosis. Additionally, PGE2 enhances cancer cell motility and metastasis by increasing MMP activity and facilitating immune evasion via IL-10 upregulation [63,64]. Beyond COX inhibition, NSAIDs also suppress VEGF expression and SOX2 oncogene activity, limiting metastatic potential [65].

Preclinical studies further demonstrate that NSAIDs enhance immune function by increasing NK cell and CTL activity, while also reducing MDSC function. For example, celecoxib, a selective COX-2 inhibitor, restores T cell activity and improves immune responses [156]. COX-2 is frequently overexpressed in cancers such as breast, bladder, and colorectal tumors, driving prostaglandin synthesis and contributing to tumor progression [66,67,157].

(2) Clinical Findings and Outcomes

Perioperative administration of NSAIDs has been shown to decrease inflammatory response and opioid consumption, potentially preserving immune function and lowering cancer recurrence risks. In breast cancer surgery, several studies reported improved disease-free survival (DFS) and OS with intraoperative NSAIDs like ketorolac or diclofenac. One study found that NSAIDs administration significantly improved DFS (HR 0.57, 95% CI 0.37–0.89, *p* = 0.01) and OS (HR 0.35, 95% CI 0.17–0.70, *p* = 0.03) [158]. Another study identified the early postoperative period (9–18 months after surgery) as a critical window for NSAID efficacy, showing reduced relapse rates during this time [159]. Additionally, multivariate analyses confirmed that ketorolac administration significantly lowered the recurrence risk after adjusting for confounders such as age, histological grade, and lymph node involvement (*p* = 0.019) [107]. However, studies in prostate cancer and NSCLC found no significant improvement in oncologic outcomes with NSAID use [160,161,162]. This suggests that the benefits of intraoperative NSAIDs may depend on the cancer type and other perioperative factors.

In contrast to perioperative use, long-term NSAID administration, particularly daily low-dose aspirin, has demonstrated preventive effects against certain cancers, such as colorectal and lung cancer [163,164]. Prolonged NSAID use has been associated with improved survival in colorectal and breast cancers [165,166,167,168,169]. However, the use of NSAIDs after diagnosis showed no survival benefit in breast cancer patients [170].

A meta-analysis of 16 studies found the evidence for NSAIDs reducing cancer recurrence to be inconclusive [171]. Additionally, an RCT investigating perioperative NSAIDs in colorectal cancer was terminated early due to the global withdrawal of rofecoxib, with the interim data showing no improvement in prognosis or recurrence prevention [172]. These mixed results highlight the need for further research to clarify the role of COX-2 inhibitors in cancer treatment.

#### 4.3.2. Dexmedetomidine

(1) Mechanistic and Preclinical Insights

Dexmedetomidine, an alpha-2 adrenergic agonist, is used for sedation and analgesia. Its anti-inflammatory properties can preserve immune function, potentially improving cancer outcomes. By reducing the sympathetic response to surgical stress, dexmedetomidine lowers catecholamine and stress hormone levels, which may otherwise promote tumor growth [68]. The drug also enhances immune function by increasing NK cells, B cells, and cluster of differentiation (CD) 4+ T cells, while improving CD4+/CD8+ and Th1/Th2 ratios [68,173].

Preclinical studies, however, present mixed evidence. On one hand, dexmedetomidine has been shown to promote angiogenesis and tumor growth by increasing HIF-1α and VEGF levels and activating the PI3K/AKT and MAPK pathways [70,71,72]. It also upregulates MMPs and induces MDSCs, facilitating invasion and metastasis in lung, liver, and colon cancers [73,74,75,76]. On the other hand, dexmedetomidine may inhibit metastasis by increasing miR-143-3p expression and suppressing EGF receptor activity, which limits cancer progression [69].

(2) Clinical Findings and Outcomes

The clinical impact of dexmedetomidine on cancer outcomes remains controversial. Retrospective studies have shown no significant effect on recurrence or survival in patients undergoing surgery for hepatocellular, colorectal, or breast carcinoma [73,107,174]. However, some studies suggest it may reduce postoperative complications and preserve immune function [175,176]. In NSCLC, one retrospective study linked intraoperative dexmedetomidine use with worse OS (HR 1.28, 95% CI 1.03–1.59, *p* = 0.024), while RFS remained unaffected (HR 1.18, 95% CI 0.91–1.53, *p* = 0.199) [113]. Conversely, an RCT involving elderly patients undergoing non-cardiac surgery reported no significant impact on OS (adjusted HR 0.78, 95% CI 0.53–1.13, *p* = 0.187) but found improved RFS (adjusted HR 0.67, 95% CI 0.49–0.92, *p* = 0.012) and event-free survival (adjusted HR 0.78, 95% CI 0.61–1.00, *p* = 0.047) in cancer patients [177].

#### 4.3.3. Ketamine

(1) Mechanistic and Preclinical Insights

Ketamine, an N-methyl-D-aspartate (NMDA) receptor antagonist, is widely used for anesthesia and analgesia. It exhibits anti-inflammatory properties by reducing cytokines, such as IL-6 and TNF-α, which may inhibit tumor growth [178]. However, ketamine may also impair immune surveillance by reducing NK cell activity and inhibiting lymphocyte maturation, potentially limiting the elimination of residual cancer cells after surgery [179,180,181].

Preclinical studies reveal mixed effects. In vitro, ketamine inhibits cancer cell growth and migration by reducing VEGF expression and blocking the AKT and ERK signaling pathways [82]. Conversely, some studies report that ketamine promotes cancer cell invasion by upregulating Bcl-2, an anti-apoptotic protein, particularly in breast cancer models [77].

In animal models, ketamine demonstrates both pro- and anti-tumor effects, depending on the cancer type and mechanism. It induces apoptosis via pathways such as CD69 expression in lung cancer [78], the Bax–mitochondria–caspase pathway in hepatocellular carcinoma [79], and NMDA receptor R2a in pancreatic carcinoma [80]. Additionally, ketamine inhibits ovarian cancer progression by downregulating long non-coding RNA PVT1 [81].

However, its immunosuppressive effects, including reduced NK cell activity and increased lymphocyte apoptosis, may increase metastasis risk [179,180,181]. These findings highlight ketamine’s dual role in cancer, with both anti-tumor and tumor-promoting potential in the perioperative setting.

(2) Clinical Findings and Outcomes

Clinical evidence on ketamine’s effects on cancer recurrence remains inconsistent [107,182]. Some studies suggest that ketamine may reduce postoperative inflammation by suppressing pro-inflammatory cytokines, such as IL-6 and TNF-α, potentially lowering cancer recurrence risks [183]. However, its immunosuppressive effects, including reduced NK cell activity and increased lymphocyte apoptosis, raise concerns about promoting metastasis, particularly in certain cancer types [181,183].

While ketamine may have some protective effects by inhibiting cancer cell proliferation, its impact on long-term cancer outcomes is unclear. No conclusive evidence currently supports a definitive role for ketamine in improving oncologic outcomes, underscoring the need for further large-scale clinical trials to better understand its perioperative effects on cancer recurrence and metastasis.

### 4.4. Systemic Local Anesthetics

#### 4.4.1. Mechanistic and Preclinical Insights

Local anesthetics (LAs), such as lidocaine, bupivacaine, and ropivacaine, are widely used for perioperative pain management via regional nerve blocks and intravenous administration. Beyond their analgesic effects, LAs exhibit potential anti-tumor properties by blocking voltage-gated sodium channels (VGSCs), which are overexpressed in cancers like breast, colon, and lung [184]. In addition, LAs reduce inflammatory cytokines (e.g., IL-6, TNF-α) and adhesion molecules, preserving NK cell function and promoting lymphocyte proliferation, which enhances anti-tumor immune responses [185,186,187,188].

Preclinical studies highlight both direct and indirect anti-cancer mechanisms of LAs [189,190]. Indirectly, LAs reduce the neuroendocrine response to surgical stress, preserving immune function and reducing the need for opioids and volatile anesthetics, both of which have been linked to increased cancer recurrence. In breast and lung cancer models, lidocaine and ropivacaine decrease IL-6, TNF-α, intercellular adhesion molecule-1, and VEGF, thereby preventing metastasis and chemoresistance [90,91,92,93,94].

Directly, LAs block VGSCs, inhibiting cancer cell viability, proliferation, and migration in breast, prostate, and ovarian cancer models [83,84,85]. They also disrupt invadopodia formation, reducing cancer cell invasion and extracellular matrix degradation [95,96]. Additionally, LAs downregulate VEGF and MMP-9, inhibiting angiogenesis and metastasis [89]. Beyond ion-channel mechanisms, lidocaine induces apoptosis and inhibits proliferation in cervical, hepatocellular, and breast cancer models through ion-channel-independent pathways [86,87,88].

#### 4.4.2. Clinical Findings and Outcomes

The clinical impact of LAs on cancer recurrence is still under investigation. Early retrospective studies suggested that regional anesthesia, including LAs, could reduce cancer recurrence by modulating the stress response and supporting immune function [191]. However, more recent studies have provided mixed results. A Cochrane review concluded that the current evidence is insufficient to confirm a significant reduction in cancer recurrence with regional anesthesia [6,123,192].

Recent studies have shifted focus to the direct anti-tumor and anti-inflammatory effects of LAs, particularly through intravenous or peritumoral administration. In pancreatic cancer, intravenous lidocaine infusion was associated with improved short-term OS (HR 0.616, 95% CI 0.290–0.783, *p* = 0.013) and higher 1- and 3-year survival rates compared to the non-lidocaine group (68.0% vs. 62.6%, *p* < 0.001; 34.1% vs. 27.2%, *p* = 0.011) [122]. However, no significant difference in DFS was observed (HR 0.913, 95% CI 0.821–1.612, *p* = 0.316). In early-stage breast cancer, peritumoral lidocaine injection before surgery was associated with improved DFS (HR 0.74, 95% CI 0.58–0.95, *p* = 0.017) and OS (HR 0.71, 95% CI 0.53–0.94, *p* = 0.019) [193].

Several ongoing RCTs aim to further clarify the oncologic benefits of intravenous lidocaine. The VAPOR-C trial (NCT04316013) is investigating its effects on lung and colorectal adenocarcinoma outcomes, with results expected by 2025. Additionally, the NCT02786329 trial focuses on recurrence rates following colorectal cancer surgery.

### 4.5. General vs. Regional Anesthesia

#### 4.5.1. Mechanistic and Preclinical Insights

General anesthesia, using volatile agents, such as isoflurane and sevoflurane, triggers a stress response by increasing cortisol and catecholamine levels, which suppress NK cell and CTL activity [5,10,194]. This response promotes the release of immunosuppressive cytokines (IL-4, IL-10) and elevates pro-inflammatory markers, like IL-6 and VEGF, creating a tumor-supporting microenvironment.

In contrast, regional anesthesia, including epidural and spinal anesthesia, mitigates these effects by reducing catecholamine release, preserving immune function, and maintaining NK cell activity [5,10,194]. Regional anesthesia also lowers IL-6 and VEGF levels, preventing the formation of a pro-tumor environment. Furthermore, it supports inflammatory balance by reducing pro-inflammatory cytokines and supporting anti-inflammatory pathways.

Preclinical studies demonstrate that regional anesthesia can reduce the need for both inhaled anesthetics and opioids, helping to preserve immune function and potentially inhibit cancer progression [195,196]. In breast cancer models, combining propofol with paravertebral block has been linked to decreased levels of VEGF-C, TGF-β1, and fibroblast growth factors, which may limit metastasis [98]. Similarly, in prostate cancer models, spinal anesthesia combined with general anesthesia reduced the perioperative stress response and lowered CTCs [97].

#### 4.5.2. Clinical Findings and Outcomes

In oncologic surgeries, general anesthesia is often essential for safety and feasibility, so most studies compare general anesthesia alone with general anesthesia plus regional anesthesia. While retrospective studies and meta-analyses suggest that regional anesthesia may modulate the stress response and support immune function, its impact on long-term cancer outcomes remains inconclusive.

Several studies report potential OS benefits when regional anesthesia is combined with general anesthesia. One study observed a significant improvement in OS with epidural-supplemented anesthesia compared to general anesthesia alone (HR 0.72, 95% CI 0.55–0.94, *p* = 0.01) [132]. A meta-analysis further supported these findings, demonstrating an OS advantage (HR 0.84, 95% CI 0.74–0.96, *p* = 0.013), particularly in colorectal cancer (HR 0.65, 95% CI 0.43–0.99, *p* = 0.045) [197]. However, these studies found no significant benefit for RFS. In bladder cancer, a retrospective study showed improved OS with regional anesthesia, though the result was not statistically significant (5-year survival: 96.3% vs. 87.5%, *p* = 0.099) [125]. In contrast, another study on colorectal liver metastases found that regional anesthesia, specifically epidural analgesia, was associated with improved RFS (5-year RFS: 34.7% vs. 21.1%, HR 0.74, 95% CI 0.56–0.95, *p* = 0.036), although no significant difference in OS was observed (HR 0.72, 95% CI 0.49–1.07, *p* = 0.102) [198].

RCTs provide similarly mixed results. In breast cancer surgery, trials comparing general anesthesia with or without a thoracic paravertebral block found no significant differences in recurrence rates [105,106]. In lung cancer, combining epidural anesthesia with general anesthesia did not improve RFS, cancer-specific survival, or OS [112]. RCTs in colorectal and prostate cancer surgeries also failed to show significant oncological benefits from adding regional anesthesia [124,199]. Similar findings were observed in trials involving major non-cardiac thoracic or abdominal surgeries [200].

However, some positive findings exist. In high-risk non-muscle invasive bladder cancer, spinal anesthesia was associated with improved RFS compared to general anesthesia (5-year RFS: 77.6% vs. 50.3%, HR 2.35, *p* = 0.016), though this benefit was not observed in low- to intermediate-risk patients [126].

In summary, while preclinical and retrospective evidence suggests that regional anesthesia may offer immunomodulatory and anti-inflammatory benefits, clinical results remain inconsistent. Regional anesthesia reduces stress hormones and preserves immune function, but these effects alone may not be sufficient to prevent recurrence or metastasis. Additionally, low concentrations of LAs at micro-metastatic sites could limit their anti-tumor efficacy [8].

## 5. Balancing Short-Term Recovery and Long-Term Oncologic Outcomes in Anesthetic Management

While much attention has been given to the long-term oncologic implications of anesthetic agents, their role in ensuring intraoperative safety and optimizing postoperative recovery is equally significant. Anesthetic regimens with potential anti-cancer effects exhibit varying impacts on intraoperative safety and postoperative recovery, highlighting that their use may not be without trade-offs. Different anesthetic techniques variably affect hemodynamic stability, postoperative pain management, and recovery-related outcomes, which must be carefully weighed against their potential effects on cancer recurrence and metastasis.

For example, propofol-based TIVA reduces postoperative nausea and vomiting and provides better patient comfort compared to volatile anesthetics, thereby enhancing overall recovery quality [201,202,203]. However, TIVA has been associated with an increased risk of surgical complications and cardiovascular instability, posing challenges in vulnerable populations, such as children, the obese, and the elderly [203,204]. Additionally, issues like propofol infusion syndrome and difficult intravenous access can further complicate its administration. Dexmedetomidine reduces postoperative pain intensity, perioperative opioid consumption, and associated complications [205,206]. It also appears to enhance recovery quality and may lower the risk of chronic postoperative pain, although the supporting evidence is of moderate to low reliability [207]. However, it poses risks of significant hemodynamic instability, such as bradycardia and hypotension, requiring careful assessment of patient-specific risks [207,208]. Regional anesthesia provides effective analgesia and reduces systemic stress responses, enhancing the postoperative recovery process. It is linked to lower opioid requirements and a reduced incidence of complications, like nausea and vomiting, while also improving patient-reported outcomes, like quality of life and time to return to work [209,210,211]. However, its invasive nature carries procedural risks, including nerve damage or infection, which must be carefully considered in certain patient populations [212,213].

Ethical considerations further complicate anesthetic decisions, particularly in resource-limited settings. Limited access to target-controlled infusion for precise TIVA dosing or ultrasound guidance for safe nerve block administration may compromise anesthetic precision and safety, potentially affecting patient outcomes. Cost differences between volatile anesthetics and TIVA, as well as the expense of newer agents, like dexmedetomidine or advanced regional techniques, often influence clinical choices. Additionally, intravenous lidocaine shows promise for its anti-cancer effects but remains off-label for this indication, highlighting the need for further research.

By integrating clinical and ethical perspectives, anesthesiologists can develop comprehensive perioperative strategies that enhance short-term recovery and patient well-being while maintaining vigilance over long-term oncologic outcomes. Tailoring anesthetic protocols to individual patient needs, considering immediate clinical benefits and potential oncologic risks, is essential to optimizing cancer care in diverse healthcare settings.

## 6. Insights from Current Trials and Proposed Research Directions

Recent clinical trials aim to clarify how different anesthetic techniques influence cancer recurrence rates in surgical patients, with a specific emphasis on inhaled anesthetics and TIVA. The VAPOR-C trial (NCT04316013) is investigating the long-term effects of propofol-based TIVA versus sevoflurane on DFS in patients undergoing surgery for lung and colorectal cancers [214]. The early findings indicate a potential advantage of TIVA, though full results are still pending. Similarly, the GA-CARES trial (NCT03034096) is evaluating the impact of various anesthetic agents on all-cause mortality across various cancer types [215]. The GAS-TIVA trial (NCT06330038) focuses specifically on NSCLC, comparing RFS between inhaled anesthetics and TIVA [216]. These trials are expected to offer valuable insights into refining anesthetic approaches to enhance cancer outcomes.

In addition, emerging molecular mechanisms, such as ferroptosis and autophagy, present exciting opportunities for advancing cancer research. Ferroptosis, a regulated form of cell death driven by iron-dependent lipid peroxidation, is distinct from apoptosis and necrosis [217]. It is primarily regulated by key molecules, such as glutathione peroxidase 4 (GPX4), which prevents lipid peroxidation and subsequent cell death. Conversely, autophagy serves a dual purpose. It helps cells survive under stress but can also initiate ferroptosis through ferritinophagy, a process that releases free iron and promotes the generation of reactive oxygen species. These pathways offer promising therapeutic targets, particularly in the context of anesthetic management. Anesthetic agents, such as propofol and dexmedetomidine, have shown potential in modulating these mechanisms [218]. Propofol is known to influence oxidative stress and autophagic activity, while dexmedetomidine may counteract ferroptosis by upregulating GPX4 expression. Investigating these interactions could provide critical insights into how perioperative anesthetic choices affect cancer cell behavior, potentially shaping new therapeutic strategies that integrate anesthetic techniques with molecular interventions to improve oncologic outcomes.

## 7. Conclusions

This review highlights the influence of anesthetic techniques on cancer recurrence and metastasis, underscoring the clinical significance of perioperative management. Inhaled anesthetics and opioids are associated with immune suppression and pro-inflammatory responses, whereas propofol-based TIVA and regional anesthesia demonstrate potential benefits in preserving immune function and reducing inflammation. Despite these promising findings, inconsistencies across cancer types and study designs indicate the need for more comprehensive research to guide clinical practice.

Personalized anesthetic strategies, informed by both ongoing large-scale trials and emerging mechanistic insights, could play a crucial role in improving oncologic outcomes. Such approaches may optimize perioperative care, balancing immediate surgical safety with long-term cancer control, ultimately enhancing patient survival and quality of life.

## Figures and Tables

**Table 1:** Summary of anesthetic techniques and their mechanistic impact on cancer progression.

Anesthetic Technique	Mechanisms Affected	Molecular Markers	Impact on Cancer Progression	References (Related Impacts on Cancer Progression)
Inhaled anesthetics	↑ Stress response, ↑ inflammatory response, ↓ immune function	↑ IL-6, ↑ TNF-α, ↓ NK cells, ↑ VEGF, ↑ HIF-1α, ↑ MMPs, ↑ CTCs	↑ CTC survival, ↑ angiogenesis, ↑ tumor cell migration	[7,44], [45,46], [47,48,49,50,51,52]
Propofol-based TIVA	↓ Stress response, ↓ inflammatory imbalance, ↑ immune function	↓ IL-6, ↓ PGE_2_, ↑ NK cells, ↓ VEGF, ↓ HIF-1α, ↓ MMPs, ↓ CTCs	↓ CTC survival, ↓ angiogenesis, ↓ tumor cell migration	[53], [45,53,54], [53,55,56,57]
Opioids	↑ Stress response, ↑ inflammatory response, ↓ immune function, ↑ MOR activation	↑ IL-6, ↓ NK cells, ↓ CTLs ↑ VEGF	↑ Tumor proliferation, ↑ angiogenesis, ↑ tumor cell migration	[9,58,59], [58,60], [58,59,61,62]
NSAIDS	↓ Inflammatory response	↓ COX2, ↓ PGE_2_, ↓ IL-10, ↓ VEGF, ↓ MMPs, ↓ MDSCs	↓ Tumor proliferation, ↓ angiogenesis, ↓ tumor cell migration	[63,64,65], [63,64], [63,64,66,67]
Dexmedetomidine	↓ Stress response, ↓ inflammatory imbalance,	↑ VEGF, ↑ HIF-1α, ↑ MMPs, ↓ EGFR, ↑ MDSCs	↓ Tumor growth, (dual effects based on dose and context) ↑ angiogenesis, ↑ tumor cell migration	[68,69], [70,71,72], [73,74,75,76]
Ketamine	↓ Inflammatory response, modulates NMDA receptors	↓ IL-6, ↓ TNF-α, ↓ NK cells, ↓ VEGF, ↑ ROS	↓ Tumor growth (mixed impact)	[77,78,79,80,81], [82]
Local anesthetics	↓ Stress response, ↓ inflammatory response, ↑ immune function, blocks sodium channels, direct cytotoxicity	↓ IL-6, ↓ TNF-α, ↓ NK cells, ↓ VEGF, ↓ MMPs, ↓ CTCs	↑ Apoptosis, ↓ angiogenesis, ↓ tumor cell migration	[83,84,85,86,87,88], [89], [89,90,91,92,93,94,95,96]
Regional anesthesia	↓ Systemic stress response	↓ IL-6, ↓ NK cells, ↓ VEGF, ↓ CTCs	↓ CTC survival, ↓ angiogenesis, ↓ tumor cell migration	[97], [97,98], [97,98]

TIVA: total intravenous anesthesia; NSAIDs: non-steroidal anti-inflammatory drugs; VEGF: vascular endothelial growth factor; HIF-1α: hypoxia-inducible factor-1 alpha; CTC: circulating tumor cell; MOR: mu-opioid receptor; PGE_2_: prostaglandin E_2_; MDSC: myeloid-derived suppressor cell; NK Cell: natural killer cell; NMDA: N-methyl-D-aspartate.

**Table 2:** Summary of clinical evidence comparing anesthetic techniques.

Cancer Type	Anesthetic Techniques	Findings	Study Type	References
Breast	TIVA (vs. Inhaled)	Improved OS	Retrospective	[99]
		Improved RFS	Retrospective	[100]
		No difference in OS and RFS	Retrospective	[101,102,103]
		Decreased locoregional recurrence	Retrospective	[104]
	Regional (vs. General)	No difference in recurrence rate	RCT	[105]
		No difference in recurrence rate and OS	RCT	[106]
	Dexmedetomidine	No difference in recurrence rate	Retrospective	[107]
Lung	TIVA (vs. Inhaled)	No difference in OS and RFS	Retrospective	[108]
	Opioid	Decreased OS and RFS (in stage I)	Retrospective	[109]
		Increase in recurrence rate	Retrospective	[110]
		No difference in recurrence rate and OS	Retrospective	[111]
	Regional (vs. General)	No difference in OS and RFS	RCT	[112]
	Dexmedetomidine	Decreased OS and no difference in RFS	Retrospective	[113]
Gastro- intestinal	TIVA (vs. Inhaled)	No difference in OS and RFS (in overall)	Retrospective	[114]
		Improved OS (in gastric)	Retrospective	[115]
		Improved OS (in colorectal)	Retrospective	[116]
		Improved OS and RFS (in esophageal)	Retrospective	[117]
		Improved OS (in hepatic)	Retrospective	[118]
	Opioid	Improved OS (in esophageal)	Retrospective	[119]
		No difference in OS and RFS (in esophageal)	Retrospective	[120]
		Decreased RFS and no difference in OS (in esophageal)	Retrospective	[121]
	Local anesthetics	Improved OS (in pancreas)	Retrospective	[122]
Urologic	Opioid	Decreased OS and RFS (in prostate)	Retrospective	[60]
	Regional (vs. General)	Improved OS and no difference in RFS (in prostate)	Meta-analysis	[123]
		No difference in disease-free survival (in prostate)	RCT	[124]
		No difference in OS and RFS (in bladder)	Retrospective	[125]
		Improved RFS (in bladder, esp. high-risk patients)	Retrospective	[126]
Brain	TIVA (vs. Inhaled)	No difference in OS and RFS	Retrospective	[127]
		No difference in OS and progression-free survival	Retrospective	[128]
Overall	TIVA (vs. Inhaled)	Improved OS (esp. gastrointestinal)	Retrospective	[129]
		Improved OS and no difference in RFS	Meta-analysis	[130]
		Improved OS and RFS	Meta-analysis	[131]
	Regional (vs. General)	Improved OS (esp. colorectal)	Meta-analysis	[132]

TIVA: total intravenous anesthesia; OS: overall survival; RFS: recurrence-free survival; RCT: randomized controlled trial.

## Abbreviations

CD	Cluster of differentiation
COX	Cyclooxygenase
CTC	Circulating tumor cell
CTL	Cytotoxic T lymphocyte
DFS	Disease-free survival
EGF	Epidermal growth factor
EMT	Epithelial–mesenchymal transition
ERK	Extracellular signal-regulated kinase
HIF	Hypoxia-inducible factor
IL	Interleukin
LAs	Local anesthetics
MAPK	Mitogen-activated protein kinase
MDSC	Myeloid-derived suppressor cells
MMP	Matrix metalloproteinase
MOR	μ-opioid receptor
NET	Neutrophil extracellular trap
NK cell	Natural killer cell
NMDA	N-methyl-D-aspartate
NSAIDs	Non-steroidal anti-inflammatory drugs
NSCLC	Non-small-cell lung cancer
OS	Overall survival
PGE2	Prostaglandin E_2_
PI3K	Phosphoinositide 3-kinase
RCT	Randomized controlled trial
RFS	Recurrence-free survival
TGF	Transforming growth factor
Th	Helper T cell
TIVA	Total intravenous anesthesia
TME	Tumor microenvironment
TNF	Tumor necrosis factor
Treg	Regulatory T cell
VEGF	Vascular endothelial growth factor
VGSCs	Voltage-gated sodium channels
